# Arytenoid cartilage movements are hypokinetic in Parkinson’s disease: A quantitative dynamic computerised tomographic study

**DOI:** 10.1371/journal.pone.0186611

**Published:** 2017-11-03

**Authors:** Laura Perju-Dumbrava, Ken Lau, Debbie Phyland, Vicki Papanikolaou, Paul Finlay, Richard Beare, Philip Bardin, Stephen Stuckey, Peter Kempster, Dominic Thyagarajan

**Affiliations:** 1 Department of Neuroscience, Monash Medical Center, Clayton, Victoria, Australia; 2 Department of Medical Imaging, Monash Medical Center, Clayton, Victoria, Australia; 3 Department of Surgery, Monash Medical Center, Clayton, Victoria, Australia; 4 Department of Respiratory Medicine, Monash Medical Center, Clayton, Victoria, Australia; Hudson Institute, AUSTRALIA

## Abstract

**Background:**

Voice change is one of the earliest features of Parkinson’s disease. However, quantitative studies of vocal fold dynamics which are needed to provide insight into disease biology, aid diagnosis, or track progression, are few.

**Methods:**

We therefore quantified arytenoid cartilage movements and glottic area during repeated phonation in 15 patients with Parkinson’s disease (symptom duration < 6 years) and 19 controls, with 320-slice computerised tomography (CT). We related these measures to perceptual voice evaluations and spirometry. We hypothesised that Parkinson’s disease patients have a smaller inter-arytenoid distance, a preserved or larger glottic area because vocal cord bowing has previously been reported, less variability in loudness, more voice dysdiadochokinesis and breathiness and a shortened phonation time because of arytenoid hypokinesis relative to glottic area.

**Results:**

Inter-arytenoid distance in Parkinson’s disease patients was moderately smaller (Mdn = 0.106, IQR = 0.091–0.116) than in controls (Mdn = 0.132, IQR = 0.116–0.166) (W = 212, P = 0.015, r = −0.42), normalised for anatomical and other inter-subject variance, analysed with two-tailed Wilcoxon’s rank sum test. This finding was confirmed in a linear mixed model analysis—Parkinson’s disease significantly predicted a reduction in the dependent variable, inter-arytenoid distance (b = −0.87, SEb = 0.39, 95% CI [−1.66, −0.08], t(31) = −2.24, P = 0.032). There was no difference in glottic area. On perceptual voice evaluation, patients had more breathiness and dysdiadochokinesis, a shorter maximum phonation time, and less variability in loudness than controls. There was no difference in spirometry after adjustment for smoking history.

**Conclusions:**

As predicted, vocal fold adduction movements are reduced in Parkinson’s disease on repeated phonation but glottic area is maintained. Some perceptual characteristics of Parkinsonian speech reflect these changes. We are the first to use 320-slice CT to study laryngeal motion. Our findings indicate how Parkinson’s disease affects intrinsic laryngeal muscle position and excursion.

## Introduction

Up to 89% of patients with Parkinson’s disease report speech symptoms [1]. Reduced variability of the fundamental frequency of speech has been estimated to precede the diagnosis of Parkinson’s disease by 5 years [2]. In a study of prodromal Parkinson’s disease [3], facial akinesia and perceptual voice changes measured on the Unified Parkinson’s Disease Rating Scale (UPDRS) were extrapolated back as far as 9.8 years before diagnosis, earlier than rigidity, gait deficits and limb bradykinesia [2].

Perceptually, parkinsonian speech is characterised by hypophonia (lower speech volume or intensity), dysphonia (poor voice quality), hypokinetic articulation (smaller range of articulatory movements), dysprosodia (smaller pitch variability and range), rush (a tendency for speech articulation to festinate) and hesitant and/or dysfluent speech [4] [5]. Even early in Parkinson’s disease, when articulation might not be compromised, there is smaller variability of pitch and loudness with more breathiness and roughness [1]. When patients with Parkinson’s disease are cued to speak loudly, they cannot sustain phonation as well as controls can, despite similar respiratory kinematics on inspiration [6]. The key features of this “hypokinetic dysarthria” are hypophonia and less variability in pitch and loudness. Hypokinetic dysarthria may be considered an analogue of limb hypokinesia in Parkinson’s disease [4] [6]. This lower voice intensity and smaller pitch and loudness variability are not simply the result of motor deficits: abnormal sensory processing and internal cueing are important as well [7] [8].

In addition to perceptual evaluation of voice quality, several other techniques have provided insight into vocal pathology in Parkinson’s disease: acoustic voice signal analysis, the physiologic techniques of videolaryngostroboscopy and electroglottography, and laryngeal electromyography [7, 9]. Of the techniques available to visualise vocal fold vibrations, which consist of medial and lateral movements travelling from the lower to the upper vocal fold lips (known as the mucosal wave) [10], direct imaging with videolaryngostroboscopy is the most commonly used. Indirect imaging techniques, such as electroglottography and photoglottography, are more cost-effective than visualisation techniques. However, none of these alone can quantify mucosal wave parameters.

Videolaryngostroboscopy studies have shown that as Parkinson’s disease progresses, glottic competence and vocal fold vibration are compromised, with a bowed closure configuration, phase asymmetry, aperiodicity, voice tremor and mucosal wave abnormalities [11] [12, 13]. This technique has certain drawbacks, however: it is subjective (stroboscopists require significant training to reduce variation and bias); the numerous parameter rating scales are inconsistent and have not been standardised; and it is applicable only to periodic vocal fold vibrations (because it creates a composite image averaged over several vibratory cycles, capturing at 30 frames per second). Thus, disorders characterised by aperiodicity or fluctuating frequency are inadequately visualised by videolaryngostroboscopy [10]. By contrast, high speed digital imaging can capture multiple images from a single glottal cycle at 2000–5000 frames per second and can therefore be used to observe aperiodic vibrations. However, the qualitative ratings of mucosal wave parameters obtained with high speed digital imaging are difficult to reconcile with those from videolaryngostroboscopy.

Digital kymography (and the conceptually related videokymography) coupled with high speed digital imaging allow vibration and mucosal wave parameters to be quantified through kymograms [10]. These techniques hold promise for quantitative visualisation studies of vocal cord motion abnormalities in Parkinson’s disease, but they only allow vibrations in the vocal fold surface to be visualised and give no quantitative information about low frequency movements of the arytenoid cartilages during phonation. The available evidence on movement deficits of arytenoid muscles as a cause of phonatory deficits in Parkinson’s disease is mostly indirect and preliminary. Disruption to vocal adductory postures, achieved by intrinsic muscle actions on the arytenoid cartilages, has been described, although the underlying mechanism for dysfunction remains unclear [14] [11] [13] [12]. These movements of the arytenoid cartilages, like other voluntary movements in Parkinson’s disease, are likely to be disturbed, and this disturbance is likely to be a crucial feature of the early and consistent vocal pathology in the disease.

There is a gap in the knowledge about various aspects of vocal cord motion in Parkinson’s disease and suitable techniques with which to study them. The rationale for this study is to image low frequency arytenoid movements in a new way in Parkinson’s disease, potentially unlocking another method for studying the biology of the disease, aiding in diagnosis and tracking progression.

Laryngeal imaging with CT or ultrasound could permit multiplanar motion analysis of the vocal apparatus. However, conventional B-mode ultrasound and Doppler imaging ultrasound have technical shortcomings in vocal fold visualisation. High-frequency ultrasound can be combined with the Nakagami parameter (a statistical model parameter of backscattered ultrasound pulses from tissue) to determine the biomechanical properties of vocal folds [15], but this combined technique is not established enough for routine use.

In contrast to ultrasound, conventional 64 slice-CT has low temporal resolution. High-speed CT, however, has the spatial and temporal resolution to enable quantitative studies of gross arytenoid cartilage movement and glottic area during phonation. An additional advantage is that the vertical shifts/tilts in laryngeal position during phonation can be adjusted for, and areas and distances can be quantified.

Dynamic 320-slice CT permits real-time viewing of tissue structure and movement over an anatomical length of 12–16 cm by repeated data acquisition without CT table movement. It can model the dynamic processes of body structures and organs, and has been used to detect upper airway dysfunction in patients with severe, treatment-unresponsive asthma [16] and in dynamic airway collapse [17].

This is a study of laryngeal function using dynamic 320-slice CT in patients with Parkinson’s disease of less than 6 years duration. Previous studies have suggested that slight to mild hypokinetic dysarthria should be present in these patients, and we hypothesised that arytenoid cartilage movements for repeated adduction are impaired in Parkinson’s disease. We used an established battery of analytical speech and voice tests to quantify hypokinetic dysarthria, as well as dynamic 320-slice CT to measure arytenoid cartilage movements and glottic area during a vocalisation. To reduce the potential confounding effects of medication, we conducted these studies in the “off” medication state. Previous studies in which disease severity and duration were described [18] [19] led us to predict that our patients, who had relatively mild disease and no motor fluctuations or dyskinesias, were unlikely to have significant upper airways obstruction, although they might have restrictive pulmonary defects. However, another study [20] found a much higher rate of upper airway obstruction in Parkinson’s disease patients. In that study, patients were studied after levodopa had been withdrawn, which may underlie the conflicting findings [21] because pulmonary function abnormalities are sensitive to levodopa treatment [19]. In light of these studies, to determine whether arytenoid motion is confounded by respiratory complications of Parkinson’s disease, particularly upper airway obstruction, we also performed spirometry on the day of the CT scan.

Our main hypotheses were as follows. First, inter-arytenoid distances during vocalisation, as measured with 320-slice CT, are smaller in Parkinson’s disease patients than in controls, and the arytenoid cartilages are therefore hypokinetic. Second, despite arytenoid cartilage hypokinesis in Parkinson’s disease, glottic area is maintained or even greater relative to the distance because of vocal cord bowing, which has previously been shown in video endoscopic studies [12, 14]. Our secondary hypotheses were as follows. Hypokinetic arytenoid cartilage motion is reflected in perceptual measures of voice: there is less variability in loudness and more dysdiadochokinesis in Parkinson’s disease. A larger glottic area in proportion to inter-arytenoid distance is reflected in more breathiness during conversation and sustained phonation and in a reduced phonation time in Parkinson’s disease.

## Materials and methods

### Patients and recruitment

We recruited 15 patients with Parkinson’s disease and 19 healthy controls of a similar age, ranging from 57 to 86 years old, from the Movement Disorders Clinic at Monash Medical Centre. Diagnoses of idiopathic Parkinson’s disease conformed to the UK Brain Bank Criteria [22]. The statistical modelling accounted for sex differences, although patients and controls were not matched for sex.

Time from onset of Parkinson’s disease symptoms was no more than 6 years, with Hoehn and Yahr stage 2.5 or less. Control subjects had no neurological disorders on the basis of history and examination by a neurologist and normal basic investigations such as cerebral imaging with CT or MRI. The demographic characteristics are detailed in Table 1. We excluded patients with respiratory or laryngeal disorders and brain or head and neck cancer.

**Table 1:** Subject and voice characteristics, lung function tests.

			Parkinson’s disease	Controls	Test Statistic	P
n (F:M)			15 (5:10)	19 (8:11)	NA	.73
Mean age in years (SD)			70.3 (7.2)	70.8 (7.4)	t(32.6) = 0.17	.86
Mean disease duration in months (SD)			56.7 (25)	NA	NA	NA
Median UPDRS Part III range (IQR)			16(11.5-20.0)	NA	NA	NA
Voice Characteristics						
	Rough	0	6 (40.0%)	13 (68.4%)	W = 92	.054
		1	3 (20.0%)	4 (21.1%)		
		2	5 (33.3%)	2 (10.5%)		
		3	1 (6.7%)	0 (0%)		
		4	0 (0%)	0 (0%)		
		5	0 (0%)	0 (0%)		
	Strained	0	9 (60.0%)	16 (84.2%)	W = 114	.21
		1	6 (40.0%)	1 (5.3%)		
		2	0 (0%)	2 (10.5%)		
		3	0 (0%)	0 (0%)		
		4	0 (0%)	0 (0%)		
		5	0 (0%)	0 (0%)		
	Breathy	0	1 (6.7%)	17 (89.5%)	W = 18.5	<.001
		1	2 (13.3%)	1 (5.3%)		
		2	10 (66.7%)	1(5.3%)		
		3	2 (13.3%)	0 (0%)		
		4	0 (0%)	0 (0%)		
		5	0 (0%)	0 (0%)		
	Dysdiadochokinesis	1	4 (26.7%)	19 (100%)	W = 38	<.001
		2	9 (60.0%)	0 (0%)		
		3	2 (13.3%)	0 (0%)		
		4	0 (0%)	0 (0%)		
		5	0 (0%)	0 (0%)		
	Loudness Variability	1	3 (20.0%)	19 (100%)	W = 28.5	<.001
		2	7 (46.7%)	0 (0%)		
		3	5 (33.3%)	0 (0%)		
		4	0 (0%)	0 (0%)		
		5	0 (0%)	0 (0%)		
	Mean MPT in seconds (SD)		16.1 (4.7)	23.1 (7.3)	t(31.8) = 3.38	.002
	UPDRS item 18	0	2	19	W = 19	<.001
		1	11	0		
		2	2	0		
		3	0	0		
		4	0	0		
Respiratory Function Tests						
	Mean FEV1 % Pred (SD)		103(17)	104(24)	F(1,30) = 1.86	.18
	Mean FVC % Pred (SD)		108(16)	115(17)	F(1,30) = 0.86	.36
	Mean VC % Pred (SD)		109(16)	115(17)	F(1,30) = 0.91	.35
	Mean FER % Pred (SD)		74(6)	71(11)	F(1,30) = 0.96	.34
	Mean PEF % Pred (SD)		112(29)	111(19)	F(1,30) = 0.15	.70
	Mean MIP % Pred (SD)		61(19)	71(25)	F(1,30) = 0.44	.51
	Mean MEP % Pred (SD)		73(18)	87(30)	F(1,30) = 0.19	.67
	Mean Pack.Yrs (SD)		6.1(13.4)	25.9(25.8)	t(28.2) = 2.89	.007

### Experimental design

The investigators performing voice assessment, lung function testing and laryngeal CT were blinded to clinical information. Patient consent was obtained according to the Declaration of Helsinki and the study was approved by the Research Ethics Committee of Monash Health (Application # 11230B). Parkinson’s disease patients were evaluated in defined medication ‘off’ states after overnight withholding of usual Parkinson’s disease medications.

As there is no literature on quantitative CT for vocal cord movements in humans, we could not power the study based on previous estimates. We recruited subject numbers similar to moderately sized studies of vocal cord function in Parkinson’s disease.

### Perceptual voice assessment

The voice examination battery followed standard voice evaluation guidelines [23]. It comprised the prolongation of the vowel /a/, the fast repetition of /i/ for 5 seconds and reading aloud of a standard phonetically balanced passage, ‘The Rainbow Passage’ [24]. For the perceptual evaluation, we used the Perceptual Voice Profile, an auditory perceptual rating tool [25]. This involves rating 16 parameters of voice according to pitch, loudness and quality on a scale of severity: normal (0), slight (1), mild (2), moderate (3), moderate-severe (4), and severe (5) for each parameter. For the purpose of this study, we analysed measures of breathiness, strain, roughness and reduction in loudness variability. The evaluations were made by investigator DP, a speech pathologist trained in this evaluation with a 90% intra and inter-rater reliability established in previous studies [26] [27]. The perceptual evaluations of audio samples were independently repeated by another rater (an experienced voice clinician fully trained and calibrated in use of the Perceptual Voice Profile) who was blinded to whether or not a subject had Parkinson’s Disease. To increase our confidence in the repeatability of perceptual voice assesments, both raters scored audio samples of a further 20 subjects, 10 with PD and 10 normal controls. Only subject age and gender were provided to the raters, and the order of presentation was randomised. The two raters also judged vocal diadochokinesis, according to the same features as articulatory diadochokinesis tasks: degree of impaired regularity, speed and precision of discrete repetitions (i.e., a score of 0 is normal and 5 severely impaired). A higher score is therefore increased dysdiadochokinesis referring to slower, faster or more irregular repetitions or imprecision of /i/ phonations than normal.

### Lung function testing

Standard measurements were obtained during spirometry, including forced vital capacity (FVC), forced expiratory volume in 1 second (FEV1) and forced expiratory ratio (FER). These measures distinguish between normal, obstructive and restrictive lung function abnormality. Maximal inspiratory pressure (MIP) and maximal expiratory pressure (MEP) were also measured; these represent a measure of respiratory muscle strength. All of the above were conducted according to American Thoracic Society guidelines [28]. Smoking pack years is the number of packs smoked per day times the number of smoking years.

### Analysis of repeated vocalisation by 320-slice CT

Vocal fold movements during phonation were imaged using dynamic 320-slice volume CT (Aquilion One CT, Toshiba Medical Systems, Tokyo, Japan). This scanner covered an anatomical volume over a length of 16cm in the Z-axis by employing 320 rows of ultra-high resolution 0.5mm detectors array for continuous data acquisition without CT table movement. Scanning parameters were 80 kV, 300–350 mA and gantry rotation 350 ms. Patients were scanned in supine positions. Each subject was trained to deliver five short, clear and fast phonations of /i/ during the scanning. Continuous CT acquisition over 5 seconds covered these phonations. Integrated CT software programs were used to obtain continuous dynamic axial, sagittal and coronal multiplanar images of the larynx, adjusted to the plane of vocal folds, which constantly change during phonation. Images were reconstructed at 100 ms/frame. Radiation doses were in a range of approximately 0.8–2.0 mSv depending on the amount of soft tissue in the neck.

**Fig 1:**
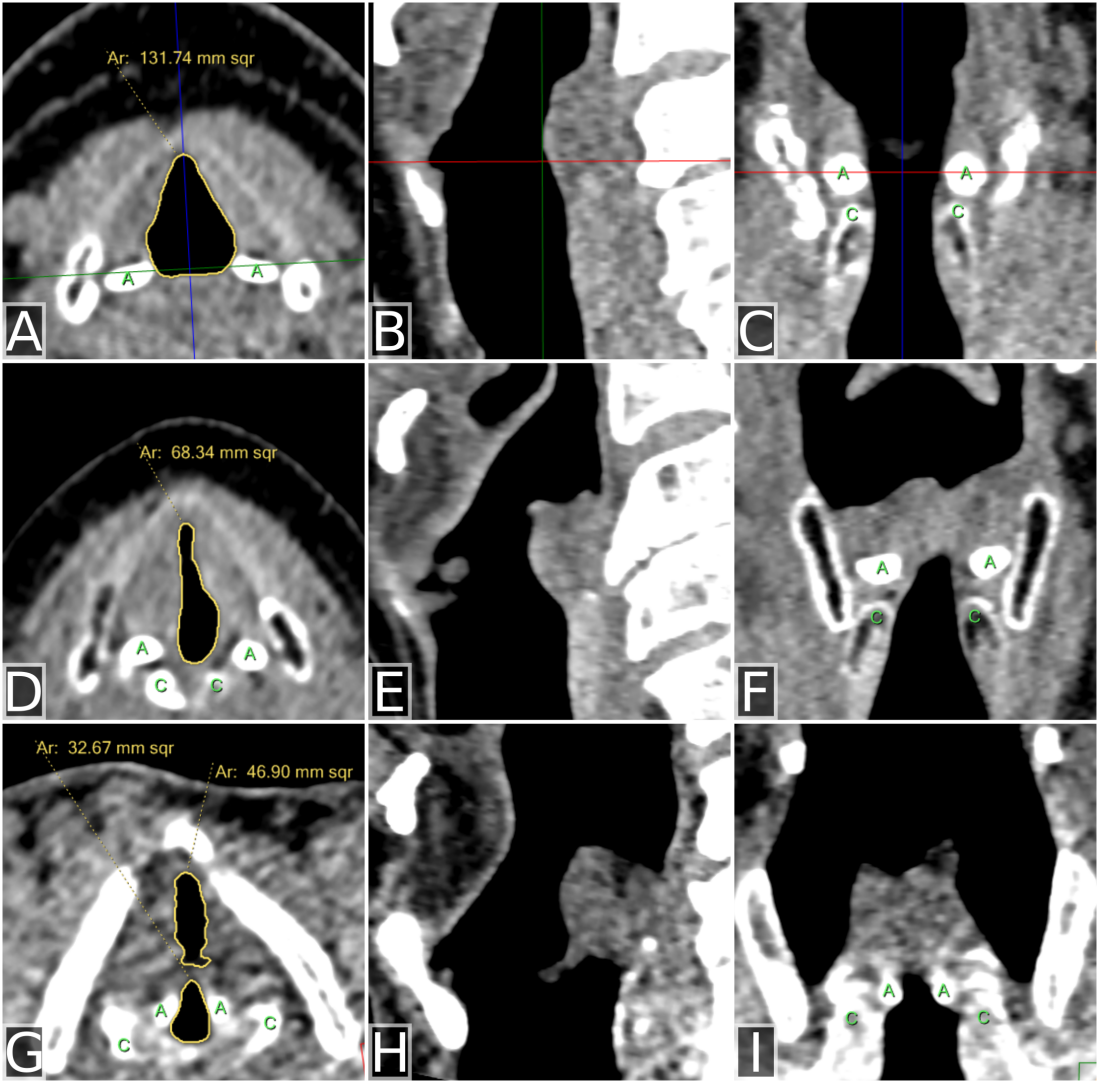
Representative glottic areas measured by automated segmentation. Each row shows a measurement taken at a single time point in a different subject. Axial, sagittal and coronal images are shown left to right. Cords apart (A, B, C), Cords towards closure (D, E, F). Hourglass deformity (G, H, I). Annotations: A indicates the arytenoid cartilage, and C, the cricoid cartilage.

Glottic area (*ga*) measurements were taken from each image at 100 ms intervals (see Fig 1). Occasionally in both controls and patients with Parkinson’s disease, an “hourglass deformity” was seen (Fig 1G, 1H and 1I). In that circumstance, we computed the areas separately then summed them.

**Fig 2:**
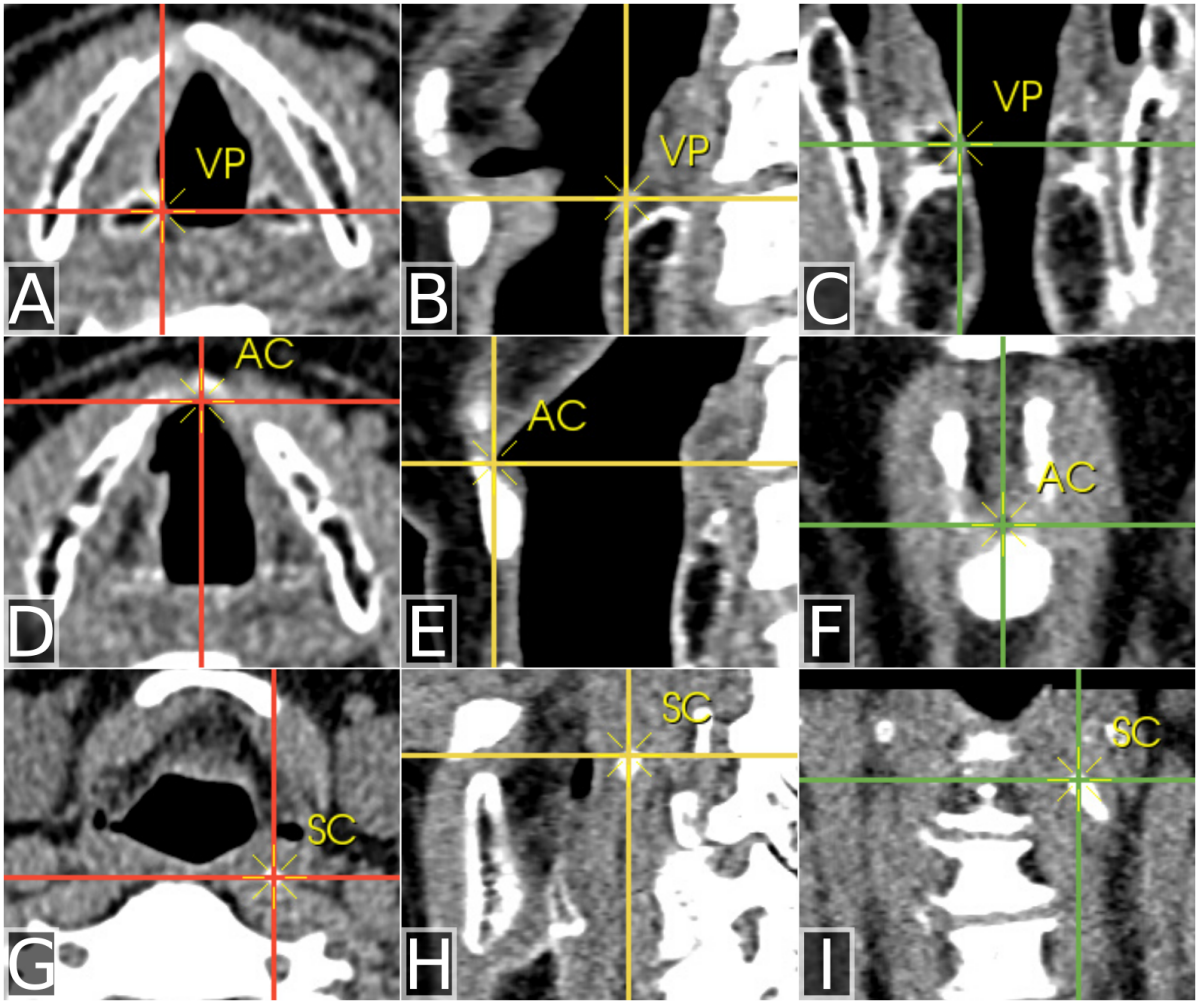
Inter-arytenoid distance. At each 100 ms interval, fiducial markers were placed on the vocal processes (VP) (A-C in the axial, sagittal and coronal planes), the anterior commissure (AC) (D-F in the axial, sagittal, and coronal planes) and finally on the superior cornua (SC) (G-I in the axial, sagittal, and coronal planes).

For arytenoid motion analysis, three fiducial markers were placed at each time point using the freely available Slicer 3D software (version 3.6.3) (http://www.slicer.org)–one on each of the vocal processes of the arytenoid cartilages, and one on the anterior commissure (Fig 2). Then two final fiducial markers were placed on the tips of the superior cornua of the thyroid cartilage. From these fiducial marker coordinates, we computed inter-arytenoid distance (*iad*) between the vocal processes every 100 ms and the inter-cornu distance (*icd*).

**Fig 3:**
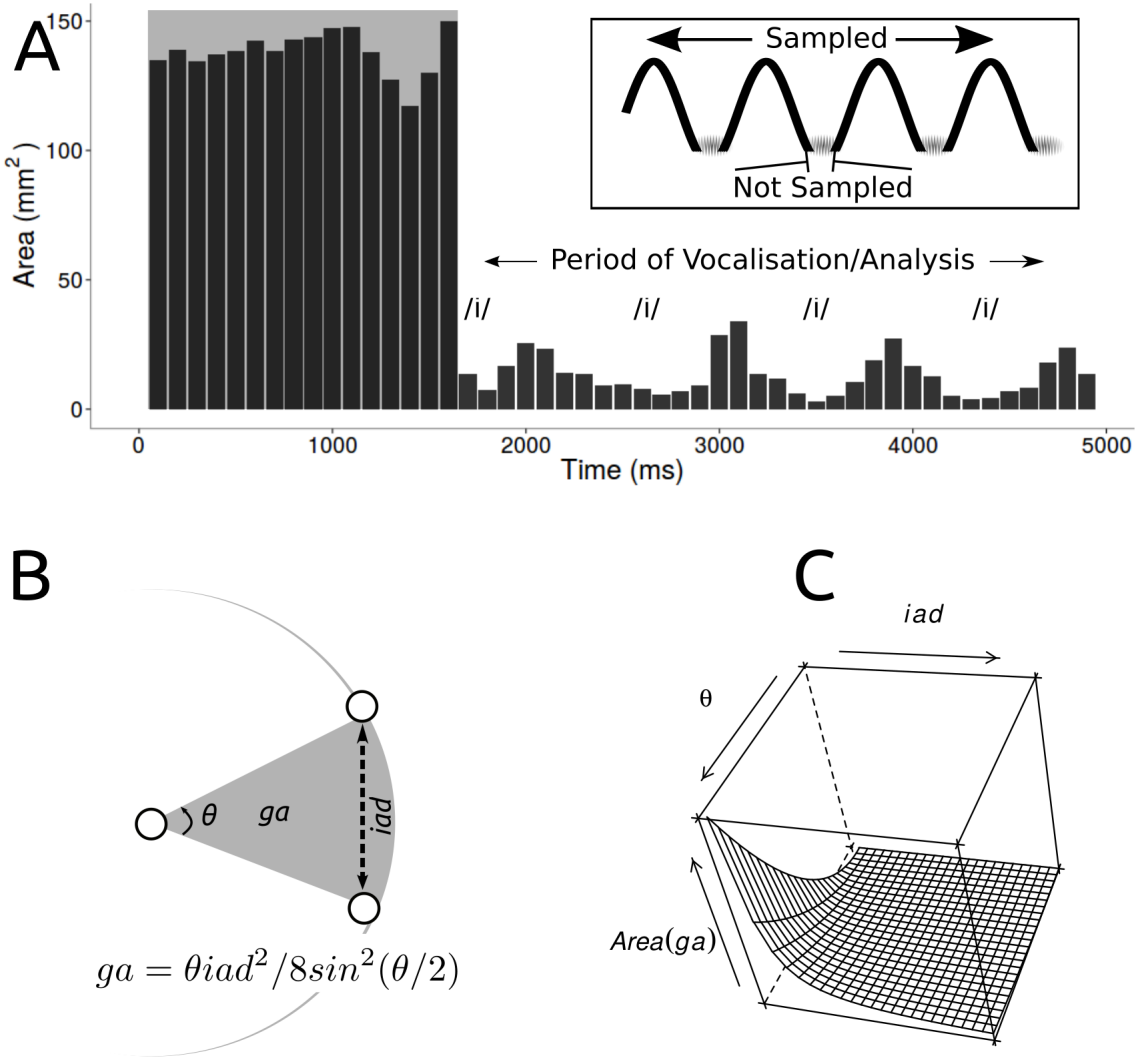
Glottic area during vocalisation. A. A representative subject. The shaded area, removed in pre-processing, corresponds to a variable time before the subject has begun vocalising. B. Relationship between glottic area (*ga*) and inter-arytenoid distance (*iad*) and C. Graphical representation. Arytenoid movement may be described as a circle centred at the anterior commissure: the relationship is quadratic. As the inter-arytenoid distance and the angle between the vocal folds increases glottic area also increases. Despite finding smaller inter-arytenoid distance during vocalisation in Parkinson’s disease, we did not find the expected reduction in the glottic area, suggesting the vocal folds are bowed during vocalisation in Parkinson’s disease.

Pre-processing: The pre-vocalisation period (shaded area in Figs 3A, 1A, 1B and 1C was removed from all data prior to analysis.

#### Glottic area

**Fig 4:**
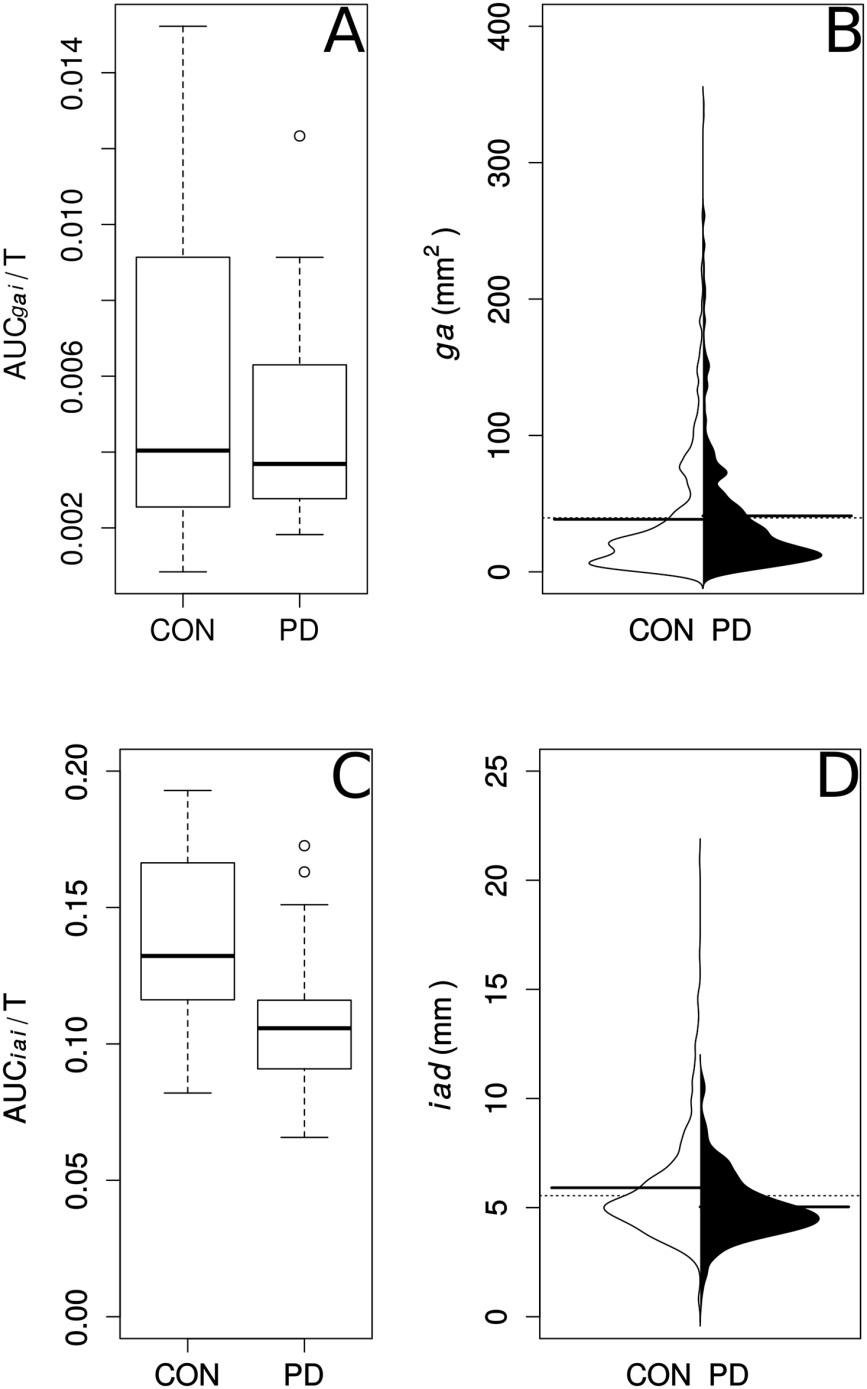
Glottic area and inter-arytenoid distance during vocalisation in Parkinson’s disease and controls. A. The area under the curve of glottic area, normalised to the superior inter cornu distance (AUC*gai*), divided by articulation time (T) is not significantly different between Parkinson’s Disease (PD) and normal patients (CON). B. Beanplot of raw glottic area measures in Parkinson’s disease and normal patients showing no significant difference in the means. C. For inter-arytenoid distance, the area under the curve of raw measurements normalised to the superior inter-cornu distance (AUC*iai*), divided by the articulation time (T) is significantly smaller in Parkinson’s disease compared with controls. D. Beanplot of raw inter-arytenoid distance measures showing the mean in Parkinson’s disease patients is lower than in controls.

We used two analytic approaches. In the first, we accounted for anatomical differences amongst patients by dividing *ga* by *icd*, giving us an index, *gai* for the vocalisation period. We plotted *gai* over time and computed the area under the curve (AUC*gai*) by the trapezoidal method which estimates area under a curve by dividing it into a number of trapezoids. It was chosen because it is accurate where periodic functions are concerned and we made the assumption that vocal cord movement in this task would approximate a sinusoidal function. To account for different vocalisation periods amongst patients we divided AUC*gai* by the length of the vocalisation period (T). This gave a single index value for each subject, normalised for duration of vocalisation and anatomical factors, the comparison between patients with Parkinson’s disease and controls displayed as a grouped boxplot (Fig 4A).

In the second approach, we used linear mixed effects analysis of the relationship between the condition (Parkinson’s disease/Control) and the raw individual area measures over the vocalisation period, considering them as repeated measures. This more powerful analysis takes into account variations between and within subjects (anatomical factors, vocalisation period).

#### Inter-arytenoid distance

We analysed inter-arytenoid distance in a similar fashion. First, we divided *iad* by *icd*, giving an index, *iai*. Again we accounted for individual subject differences in vocalisation period, by dividing the area under the curve (AUC*iai*) by T. The comparison of this index between Parkinson’s disease and Control groups is displayed as a grouped boxplot (Fig 4C).

Then we used linear mixed effects analysis of the relationship between the condition (Parkinson’s disease/Control) and the raw individual *iad* measures over the vocalisation period, considering them as repeated measures.

### Statistical analysis

For all statistical tests, we used an alpha level of 0.05. To compare subject characteristics, we used Fishers exact test for counts and the Student’s t test for continuous variables. Statistical analysis and graphics were performed using the R statistical and graphing environment [29] and nlme [30].

To compare perceptual voice assessments we used the Wilcoxon’s rank sum test for scale-derived ordinal data and for MPT, the Student’s t test. To assess inter-rater reliability in perceptual voice measures for breathiness, strained quality, roughness, dysdiadochokinesis and loudness variability, we used weighted Cohen’s kappa (squared weights) on scores of perceptual assessments made by two raters in a larger group of 54 subjects (25 with PD and 29 controls), a subset of which comprised the subjects in this study.

To analyse lung function data, we performed two-way analysis of variance (ANOVA) to compare the main effects of disease status (Parkinson’s disease/Control) and smoking status (Smoker/Non-smoker) and their interaction on the predicted value of each lung function parameter.

For glottic area and inter-arytenoid distance, we compared respectively AUC*gai*/T and AUC*iai*/T between patients with Parkinson’s disease and controls with the two-tailed Wilcoxon’s rank sum test. A series of Spearman rank-order correlations were conducted in order to determine if there were any relationships between the AUC*iai*/T and those perceptual measures we found to be significantly different between Parkinson’s disease patients and controls (breathiness, dysdiadochokinesis, loudness variability). A Pearson product-moment correlation coefficient was computed to assess the relationship between AUC*iai*/T and MPT. In the linear mixed effects analysis, sex and condition (Parkinson’s disease/Control) were fixed effects and subject was the random effect. The raw individual *iad* measures were the dependent variable. For model comparisons, see supporting information S1 File.

In the analyses of glottic area and inter-arytenoid distance, there was no significant difference in number of measured time points between patients with Parkinson’s disease (N = 15, M = 34.2, SD = 2.60) and controls (N = 19, M = 37.3, SD = 7.02), t (23.9) = 1.79, P = 0.087, 95% CI [−0.48, 6.72], d = 3.11.

## Results

Subject characteristics, perceptual voice assessment and lung function tests are summarised in Table 1.

### Perceptual and respiratory measures

We found no significant differences between Parkinson’s disease patients and controls in perceptual measures of voice for roughness and strained quality. Breathiness in patients with Parkinson’s disease (Mdn = 2) was greater than in controls (Mdn = 0) with r = 0.81. Dysdiadochokinesis in patients with Parkinson’s disease (Mdn = 2) was greater than in controls (Mdn = 1) with r = 0.75. Reduction in loudness variability in patients with Parkinson’s disease (Mdn = 2) was greater than in controls (Mdn = 1) with r = 0.80. MPT was significantly smaller in patients with Parkinson’s disease with r = –0.52. For the UPDRS Part III, Item 18 (speech) score, patients with Parkinson’s disease (Mdn = 1) scored higher than controls (Mdn = 0), r = 0.86. The reliability of these perceptual measures (See supporting information S2 File) was assessed as strong to almost perfect by Cohen’s kappa—for breathiness, 0.88, 95% CI [0.66, 0.95], P < 0.001; for strained quality, 0.92, 95% CI [0.79, 0.98], P < 0.001; for roughness, 0.99, 95% CI [0.93, 1.00], P < 0.001; for loudness variability, 0.91, 95% CI [0.80, 0.96], P < 0.001; and for dysdiadochokinesis, 0.93, 95% CI [0.84, 1.00], P < 0.001.

Baseline spirometry and respiratory muscle strength measures did not differ between the groups after adjusting for smoking (See supporting information S3 File).

Smoking (pack-years) was significantly lower in the patients with Parkinson’s disease compared with controls (Table 1).

### Glottic area in Parkinson’s disease does not differ from controls

By two approaches, we found that glottic area during vocalisation task is not significantly different between patients and controls. Fig 4 compares AUC*gai*/T between patients with Parkinson’s disease and controls. This measure was similar in patients with Parkinson’s disease (Mdn = 0.0037, IQR = 0.0028–0.0063) and controls (Mdn = 0.0040, IQR = 0.0026–0.0091) with W = 153, P = 0.732, r = 0.06.

Fig 4 compares the distributions of the raw values of glottic area, *ga*, between patients with Parkinson’s disease and controls. In our linear mixed model, the relationship between condition (Control /Parkinson’s disease) and *ga* showed significant variance in intercepts across patients, SD = 32.4, 95% CI [25.4, 41.2], *χ*^2^ (1) = 1166.24, P< 0.0001. However, condition as a fixed effect did not significantly predict *ga*, b = –0.59, SEb = 11.34, 95% CI [−23.69, 22.51], t(31) = 0.05, P = 0.96. Sex as a fixed effect also did not significantly predict *ga*, b = 21.09, SEb = 11.59, 95% CI [−2.52, 44.69], t(31) = 1.82, P = 0.07.

### Inter-arytenoid distance is smaller in Parkinson’s disease

On the other hand, during vocalisation, AUC*iai*/T (Fig 4C), in patients with Parkinson’s disease (Mdn = 0.106, IQR = 0.091–0.116) was smaller compared with controls (Mdn = 0.132, IQR = 0.116–0.166) with W = 212, P = 0.015, r = −0.42.

Fig 4D compares the distributions of the raw values of inter-arytenoid distance, *iad*, of Parkinson’s disease patients with control data. In our linear mixed model, the relationship between condition (Control/Parkinson’s disease) and *iad* showed significant variance in intercepts across patients, SD = 1.08, 95% CI [0.83, 1.40], *χ*^2^ = 429.62, P< 0.0001. Condition as a fixed effect significantly predicted *iad*, b = −0.87, SEb = 0.39, 95% CI [−1.66, −0.08], t(31) = −2.24, P = 0.032. Sex as a fixed effect did not significantly predict *iad*, b = 0.52, SEb = 0.40, 95% CI [−0.28, 1.33], t(31) = 1.32, P = 0.195.

### Relationship between inter-arytenoid distance and perceptual measures

A measure of inter-arytenoid distance, AUC*iai*/T, was modestly negatively correlated with breathiness, *r*_*s*_(32) = −0.34, P = 0.048, and modestly positively correlated with MPT, *r*(32) = 0.34, P = 0.047. There was no significant relationship between AUC*iai*/T and dysdiadochokinesis, *r*_*s*_(32) = −0.25, P = 0.157 or loudness variability, *r*_*s*_(32) = −0.30, P = 0.085.

## Discussion

Using novel 320-slice laryngeal CT, we demonstrated significant reduction in a scaled measure of inter-arytenoid distance during repeated vocalisation in early Parkinson’s disease. Linear mixed modelling confirmed that Parkinson’s disease predicts smaller raw measurements of inter-arytenoid distance with an effect size that was medium (r = −0.42).

We conclude that during a repeated phonation activity, a task requiring alternate vocal fold abduction and adduction, arytenoid cartilage movements are hypokinetic in Parkinson’s disease.

It is important to note that high frequency vibrations of the vocal cords are beyond the temporal resolution of our CT technique (Fig 3) and were not studied here. With 320-slice CT we examined movements of the arytenoid cartilages. From the movements of the arytenoids, which set the position and tension of the vocal folds, the actions of the intrinsic muscles on the laryngeal structures may be inferred and perceptual voice characteristics in PD at least partially explained. We quantified arytenoid hypokinesis in Parkinson’s disease two ways: the area under an approximately sinusoidal displacement-time curve (adjusted for anatomical factors and vocalisation time), and the sum of the displacements over the duration of vocalisation.

There was no reduction in glottic area across vocalisations between patients with Parkinson’s disease and controls (Fig 3B and 3C). In other words, both groups maintained their same degree of glottic closure for phonation across the task. However, because inter-arytenoid distance was smaller in Parkinson’s disease, the glottic area as a proportion of the inter-arytenoid distance during vocalisation was higher in patients with Parkinson’s disease than in controls. This is consistent with a bowed vocal fold closure pattern, previously described among Parkinson’s disease patients in videolaryngostroboscopic studies [14] [12] [11]. Across all subjects, there was a modest negative correlation between breathiness and positive correlation between MPT and a measure of inter-arytenoid distance, AUC*iai*/T. Consistent with our hypothesis, the larger glottic area:inter-arytenoid distance ratio (bowing) in Parkinson’s disease patients was thus reflected in greater breathiness during conversation and on sustained phonation, and shorter MPT. By contrast, there were no significant correlations between a measure of inter-arytenoid distance and reduction in loudness variability or dysdiadochokinesis, even though these measures were significantly higher in the Parkinsons disease patients compared with controls. This suggests that, contrary to our hypothesis, vocal fold dynamics other than arytenoid cartilage hypokinesis are responsible for changes in loudness variability and dysdiadochokinesis in Parkinson’s disease.

Smaller arytenoid cartilage movement during vocalisation suggests either that the abducting laryngeal muscles—principally the posterior cricoarytenoids—or the adductors, such as the lateral cricoarytenoids, are rigid, bradykinetic, or both. Our finding of more voice breathiness in Parkinson’s disease, and the inference of glottic incompetence from the relationship of glottic area to inter-arytenoid distance, suggests that the fine motor control of glottic closure, involving the lateral cricoarytenoid, the thyroarytenoid and cricothyroid, is disturbed. From this study we cannot determine if less glottic closure across repeated phonations is due to an isolated reduction in activation of one of these specific intrinsic laryngeal muscles or all adductor muscles. Previous studies have shown that all 3 muscle groups are involved in glottic closure [31] [32] [33]. Others propose that the relative contribution of each muscle is task dependent, with the thyroarytenoid and cricothyroid reported as the primary muscles active in closure shape for vocalisation [33] and therefore potentially most implicated in the bowing configuration seen in Parkinson’s disease [32] [14] [34].

Although the main findings of arytenoid hypokinesis and relative glottic incompetence in Parkinson’s disease logically explain the results of some of our perceptual voice evaluations some caution in interpretation is warranted because sex is known to affect perceptual voice abnormalities in Parkinson’s disease [35]. A limitation of our study is that Parkinson’s disease and controls were not matched for sex and there were unequal sex ratios in the two groups. We do not believe this sex imbalance affects the primary findings of arytenoid hypokinesis and relative glottic incompetence because our linear mixed effects model found no significant effect for sex or the interaction between sex and condition (Parkinson’s disease/Control)(See Supporting information S1 File). Dopaminergic treatment is unlikely to be a confounding factor [36] since the CT and the evaluations were conducted after overnight medication withdrawal in all Parkinson’s disease patients.

No parameters of respiratory function were significantly different between patients with Parkinson’s disease and controls, suggesting that our findings are not explained by altered dynamics of breathing. There was, however, a medium size reduction (r = −0.5) in the mean of MPT in patients with Parkinson’s disease, indicating air wastage related to glottic incompetence.

Alpha-synuclein aggregates have been demonstrated in the vagal nuclear complex, vagal nerve and its pharyngeal branch [37]. Moreover in Parkinson’s disease, pharyngeal muscles show atrophic fibres, fibre type grouping, and fast-to-slow myosin heavy chain transformation, indicating that they experience cycles of denervation and reinnervation [38]. According to the caudal-to-rostral topographic pattern implied by the Braak staging system, changes in the medulla occur early in the disease course, even before major motor symptoms.

Rather than medullary or cranial nerve pathology, vocal fold hypokinesis might be caused partly or entirely by striatal dopaminergic deficit. A future study of arytenoid cartilage movement before and after doses of dopaminergic medication would be required to address this question.

Controlled, quantitative studies of laryngeal function in Parkinson’s disease are scarce. A strength of our controlled study is the novel use of 320-slice dynamic CT. Although the temporal resolution of the technique does not capture high frequency vocal fold vibrations, it provides valuable quantitative information regarding positional arytenoid cartilage movement during vocalisation in Parkinson’s disease. The determinations of inter-arytenoid distance and glottic area were laborious and time consuming, but we have now developed an automated algorithm to facilitate these measures for further CT studies of vocal cord function in Parkinson’s disease [39] [40].

In summary, this study provides a new perspective on motor control of the larynx in Parkinson’s disease, offering observations about the dynamic function of the intrinsic laryngeal musculature. Refinements of our analytic approach may be used in future studies of the mechanisms, onset, and progression of vocal cord kinetic changes in Parkinson’s disease. This in turn, will potentially aid diagnosis and monitoring, and improve knowledge of the disease biology.

## Supporting information

S1 File Model comparisons. Table 1 shows linear mixed model comparisons for inter-arytenoid distance. Table 2 shows linear mixed model comparisons for glottic area.
(PDF)

S2 File Perceptual voice measures of two raters. Scores from 54 subjects including those in this study (25 with PD and 29 normal controls). NUMBER: subject ID. R1: the primary rater in this study (DP) and R2 is a second expert rater. DDK: diadochokinesis, BREATHY: breathiness, STRAIN: strained quality, ROUGH: roughness, LoudnessVar: loudness variability.
(CSV)

S3 File Spirometry. Two-way ANOVA to compare the main effects of disease status (PD/Control) and smoking status (Smoker/Non-smoker) and their interaction on the predicted value of each lung function parameter.
(PDF)

S4 File Glottic area measures. NUMBER: subject ID, Duration: duration of disease in months, AGE: age of subject in years, UPDRS: UPDRS Part III score, CONDITION: PD = Parkinson’s disease, CON = control, SEX: M = male, F = female, TimePoint, Raw_measure, AUC: area under curve, icd: inter–cornu distance, Scaled_measure: AUC/icd.
(CSV)

S5 File Inter-arytenoid measures. NUMBER: subject ID, Duration: duration of disease in months, AGE: age of subject in years, UPDRS: UPDRS Part III score, CONDITION: PD = Parkinson’s disease, CON = control, SEX: M = male, F = female, Time Point, Raw_measure, AUC: area under curve, icd: inter–cornu distance, Scaled_measure: AUC/icd.
(CSV)

S1 Appendix Abbreviations used in the text. (PDF)
