# Supplementary material for: Arytenoid cartilage movements are hypokinetic in Parkinson’s disease: A quantitative dynamic computerised tomographic study
Source: PLoS One. 2017 Nov 3;12(11):e0186611. doi: 10.1371/journal.pone.0186611 (PMC5669420; doi:10.1371/journal.pone.0186611)
Supplement: S1 File — Table 1 shows linear mixed model comparisons for inter-arytenoid distance. Table 2 shows linear mixed model comparisons for glottic area. (PDF) [file pone.0186611.s001.pdf]

Table 1: Inter-arytenoid distance: model comparisons. Model (2) was chosen

	Dependent variable = Inter-arytenoid distance		
	(1)	(2)	(3)
CONDITIONPD	−0.576	−0.868**	
	(0.635)	(0.387)	
SEXM	0.718	0.524	
	(0.517)	(0.396)	
CONDITIONPD:SEXM	−0.463		
	(0.799)		
Constant	5.454***	5.566***	5.508***
	(0.394)	(0.344)	(0.208)
Observations	1,222	1,222	1,222
Log Likelihood	−2,409.855	−2,410.022	−2,412.927
Akaike Inf. Crit.	4,831.710	4,830.045	4,831.853
Bayesian Inf. Crit.	4,862.359	4,855.586	4,847.178

Note: *p<0.1; **p<0.05; ***p<0.01

Table 2: Glottic Area: model comparisons. Model (2) was chosen

	Dependent variable = Glottic Area		
	(1)	(2)	(3)
CONDITIONPD	22.660	−0.593	
	(17.973)	(11.339)	
SEXM	36.493**	21.087*	
	(14.632)	(11.588)	
CONDITIONPD:SEXM	−36.865		
	(22.632)		
Constant	15.165	24.098**	36.872***
	(11.143)	(10.069)	(5.868)
Observations	1,238	1,238	1,238
Log Likelihood	−5,868.548	−5,869.828	−5,871.419
Akaike Inf. Crit.	11,749.090	11,749.660	11,748.840
Bayesian Inf. Crit.	11,779.820	11,775.260	11,764.200
Note:		*p<0.1; **p<0.05; ***p<0.01	
