# Supplementary material for: Arytenoid cartilage movements are hypokinetic in Parkinson’s disease: A quantitative dynamic computerised tomographic study
Source: PLoS One. 2017 Nov 3;12(11):e0186611. doi: 10.1371/journal.pone.0186611 (PMC5669420; doi:10.1371/journal.pone.0186611)
Supplement: S3 File — Two-way ANOVA to compare the main effects of disease status (PD/Control) and smoking status (Smoker/Non-smoker) and their interaction on the predicted value of each lung function parameter. (PDF) [file pone.0186611.s003.pdf]

Anova Table (Type II tests)

Response: FER

	Sum Sq	Df	F value	Pr(>F)
CONDITION	7.21	1	0.0850	0.7726
SmokingStatus	87.27	1	1.0295	0.3184
CONDITION:SmokingStatus	182.56	1	2.1535	0.1527
Residuals	2543.20	30		

Anova Table (Type II tests)

Response: FEV1

	Sum Sq	Df	F value	Pr(>F)
CONDITION	215.7	1	0.4745	0.4962
SmokingStatus	419.2	1	0.9221	0.3446
CONDITION:SmokingStatus	648.4	1	1.4264	0.2417
Residuals	13637.7	30		

Anova Table (Type II tests)

Response: VC

	Sum Sq	Df	F value	Pr(>F)
CONDITION	376.3	1	1.2951	0.2641
SmokingStatus	55.5	1	0.1911	0.6652
CONDITION:SmokingStatus	30.2	1	0.1041	0.7492
Residuals	8716.3	30		

Anova Table (Type II tests)

Response: FVC

	Sum Sq	Df	F value	Pr(>F)
CONDITION	448.7	1	1.5249	0.2265
SmokingStatus	99.3	1	0.3375	0.5656
CONDITION:SmokingStatus	13.4	1	0.0456	0.8324
Residuals	8827.9	30		

Anova Table (Type II tests)

Response: FRC

	Sum Sq	Df	F value	Pr(>F)
CONDITION	19	1	0.0148	0.9041
SmokingStatus	1679	1	1.3042	0.2625
CONDITION:SmokingStatus	219	1	0.1705	0.6826
Residuals	38626	30		

Anova Table (Type II tests)

Response: RV

	Sum Sq	Df	F value	Pr(>F)
CONDITION	49	1	0.0323	0.8586
SmokingStatus	3130	1	2.0754	0.1604
CONDITION:SmokingStatus	1327	1	0.8802	0.3559
Residuals	43735	29		

```
Anova Table (Type II tests)

Response: TLC
                        Sum Sq Df F value  Pr(>F)
CONDITION                417.5  1  1.9446 0.17376
SmokingStatus            773.4  1  3.6024 0.06769
CONDITION:SmokingStatus  107.3  1  0.4998 0.48523
Residuals               6225.9 29
---
Signif. codes:  0 '***' 0.001 '**' 0.01 '*' 0.05 '.' 0.1 ' ' 1

Anova Table (Type II tests)

Response: PEF
                         Sum Sq Df F value Pr(>F)
CONDITION                  86.9  1  0.1493 0.7019
SmokingStatus             614.0  1  1.0549 0.3126
CONDITION:SmokingStatus    20.7  1  0.0356 0.8517
Residuals               17462.4 30

Anova Table (Type II tests)

Response: MEP
                         Sum Sq Df F value  Pr(>F)
CONDITION                1844.0  1  3.0584 0.09055
SmokingStatus             291.8  1  0.4840 0.49198
CONDITION:SmokingStatus  2193.3  1  3.6378 0.06610
Residuals               18088.0 30
---
Signif. codes:  0 '***' 0.001 '**' 0.01 '*' 0.05 '.' 0.1 ' ' 1

Anova Table (Type II tests)

Response: MIP
                         Sum Sq Df F value  Pr(>F)
CONDITION                 951.3  1  2.0072 0.16686
SmokingStatus             133.1  1  0.2809 0.60003
CONDITION:SmokingStatus  1794.2  1  3.7854 0.06112
Residuals               14219.1 30
---
Signif. codes:  0 '***' 0.001 '**' 0.01 '*' 0.05 '.' 0.1 ' ' 1

Anova Table (Type II tests)

Response: MMEF
                         Sum Sq Df F value Pr(>F)
CONDITION                 266.1  1  0.3865 0.5389
SmokingStatus            1669.7  1  2.4251 0.1299
CONDITION:SmokingStatus  1468.2  1  2.1325 0.1546
Residuals               20654.6 30

Anova Table (Type II tests)
```

```
Response: TLCO
                        Sum Sq Df F value  Pr(>F)
CONDITION                749.4  1  3.8186 0.06006
SmokingStatus            164.2  1  0.8366 0.36766
CONDITION:SmokingStatus  597.8  1  3.0463 0.09116
Residuals               5887.5 30
---
Signif. codes:  0 '***' 0.001 '**' 0.01 '*' 0.05 '.' 0.1 ' ' 1
```
