# Supplementary material for: Arytenoid cartilage movements are hypokinetic in Parkinson’s disease: A quantitative dynamic computerised tomographic study
Source: PLoS One. 2017 Nov 3;12(11):e0186611. doi: 10.1371/journal.pone.0186611 (PMC5669420; doi:10.1371/journal.pone.0186611)
Supplement: S1 Appendix — (PDF) [file pone.0186611.s006.pdf]

FER- forced expiratory ratio

FEV1- Forced expiratory volume in 1 second

FVC- Forced Vital Capacity

MEP- Maximal expiratory pressure

MIP-Maximal inspiratory pressure

MPT- maximal vowel prolongation time

PEF- Peak expiratory flow

VC- Vital capacity
